# Supplementary material for: Francisella tularensis Glyceraldehyde-3-Phosphate Dehydrogenase Is Relocalized during Intracellular Infection and Reveals Effect on Cytokine Gene Expression and Signaling
Source: Cells. 2023 Feb 13;12(4):607. doi: 10.3390/cells12040607 (PMC9954481; doi:10.3390/cells12040607)
Supplement: Supplementary file 1 [file cells-12-00607-s001.zip › Supplementary Materials_2_proofs.pdf]

Supplementary Materials S2:

Francisella tularensis Glyceraldehyde-3-Phosphate Dehydrogenase Is Relocalized during Intracellular Infection and Reveals Effect on Cytokine Gene Expression and Signaling

Ivona Pavkova [1,*], Monika Kopeckova [1], Marek Link [1], Erik Vlcak [2], Vlada Filimonenko [2,3], Lenka Lecova [1], Jitka Zakova [1], Pavlina Laskova [1], Valeria Sheshko [1], Miloslav Machacek [4] and Jiri Stulik [1]

1 Department of Molecular Pathology and Biology, Faculty of Military Health Sciences, University of Defence, Trebesska 1575, 500 01 Hradec Kralove, Czech Republic;

2 Institute of Molecular Genetics of the Czech Academy of Sciences, Electron Microscopy Core Facility, Videnska 1083, 142 20 Prague, Czech Republic;

3 Institute of Molecular Genetics of the Czech Academy of Sciences, Department of Biology of the Cell Nucleus, Videnska 1083, 142 20 Prague, Czech Republic

4 Department of Biochemical Sciences, Faculty of Pharmacy in Hradec Kralove, Charles University in Prague, Akademika Heyrovskeho 1203, 500 05 Hradec Kralove, Czech Republic;

* Correspondence: ivona.pavkova@unob.cz

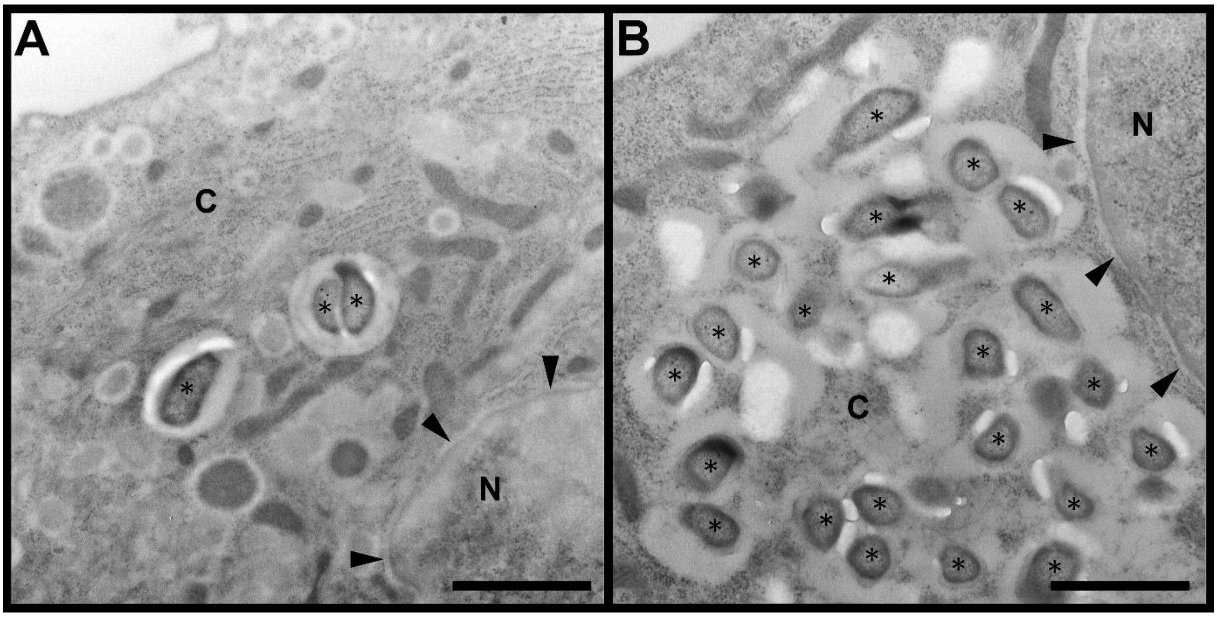

Figure S2.1. TEM micrograph of BMDM host cells containing bacteria *F. tularensis* in the cytoplasm upon 2 hours (**A**) and 12 hours (**B**) post infection. Bacteria marked with asterisk (*). Both images display nucleus (N) and cytoplasm (C) with arrowheads pointing to nuclear membrane. Scalebar = 1 μm.

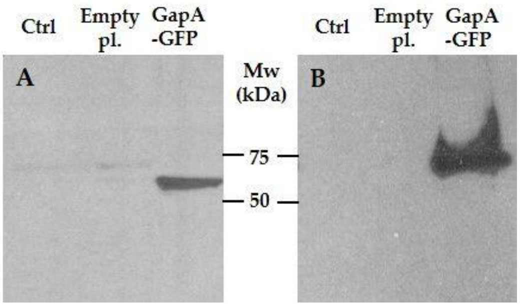

Figure S2.2. Confirmation of expression and purification of GapA-GFP from HEK293T cells. (A) HEK293T lysates transfected either with empty plasmid pEGFP-C2 (empty pl.) or pEGFP-C2::*gapA* (GapA-GFP) or non-transfected (Ctrl) were analyzed by Western blot probing for GapA. (B) HEK293T lysates transfected either with empty plasmid pEGFP-C2 (empty pl.) or pEGFP-C2::*gapA* (GapA-GFP) or non-transfected (Ctrl) were subjected to immunoprecipitation using antibodies against GFP. Samples were analyzed by Western blot probing for GapA. The bands in (A) and (B) correspond to the GapA protein (37 kDa) fused with GFP (27 kDa).

Method:

Invasion and replication of *F. tularensis* FSC200 in macrophage cell line with knock outed S100A6.

CRISPR/Cas9-mediated S100A6 knock-out in J774.1 macrophage cell line was prepared and kindly provided by Henry T., CIRI, Inserm, Lyon, France. Briefly, Cas9-expressing J774.1 murine macrophage-like cells were transduced with lentiviral particles packaged with the pKLV-U6gRNA(BbsI)-PGKpuro2ABFP plasmid (Gift from Kosuke Yusa, Addgene plasmid # 50946) with cloned gRNA targeting the gene encoding S100A6 protein. gRNA-expressing J774.1 cells were selected for 10 days in puromycin. Production of lentiviral particle was performed in 293T cells by co-transfection of the following plasmids: pMD2.G (Gift from Didier Trono, Addgene plasmid #12259), pCMVR8.74 (Gift from Didier Trono, Addgene plasmid #22036), and LentiCas9-Blast or pKLV-U6gRNA(BbsI)-PGKpuro2ABFP. Gene invalidation was verified by immunoblot analysis using the polyclonal S100A6 antibody (R&D Systems, MN, USA).

	sgRNA Target Sequence	Strand
S100A6	GGGGCTACTCACGGAGCCAA	antisense
	TCAGGCCATTGGCCTTCTCG	sense

For proliferation assay, the J774 KO/S100A6 and J774.cas were infected with *F. tularensis* subsp. *holarctica* FSC200 strain at MOI 100. To synchronize infection, the infected cells were centrifuged at 400x g for 3 min and incubated at 37°C, 5% CO_2 for 30 min. Then, the extracellular bacteria were removed by gentamicin treatment (5 μg/ml) for 30 min. At selected time point – 1, 6, 24 and 48 hours post infection, the cells were lysed with 0.1% deoxycholate and the lysates were serially diluted and plated onto McLeod agar for CFU enumeration.

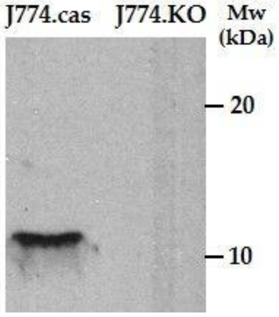

Figure S2.3. Verification of S100A6 knockout in J774.cas cells by western blot analysis using the anti-S100A6 antibody.

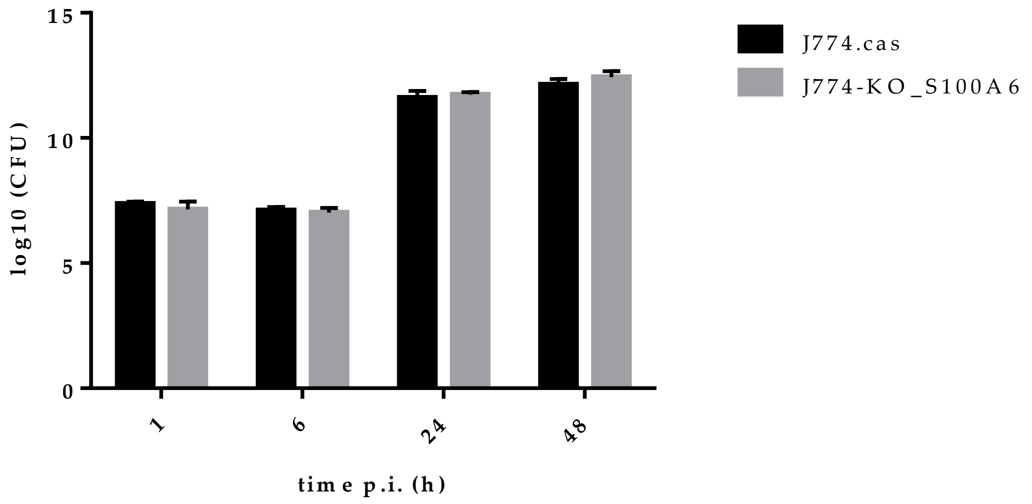

Figure S2.4. Invasion and proliferation of FSC200 wt strain in J774.cas (control) and J774.KO_S100A6 cell lines. Cells were infected at MOI of 100:1 and harvested at 1, 6, 24 and 48 h post-infection. The numbers of bacteria recovered from the cells were counted as cfu. The data are means of triplicate samples and the results shown are representatives of two independent experiments.

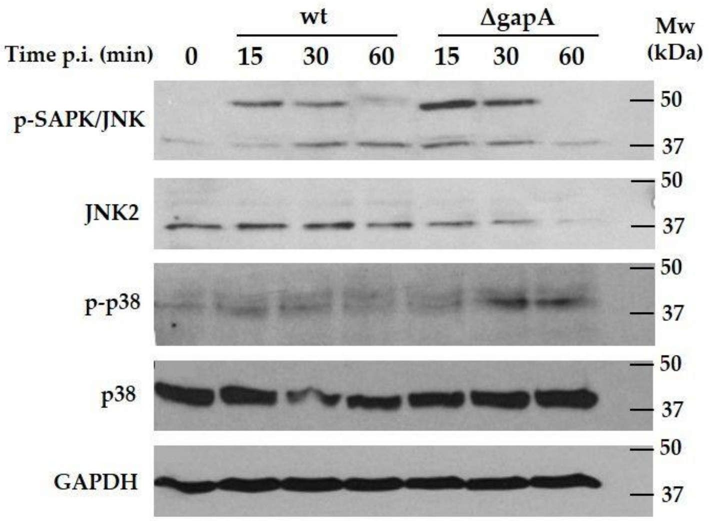

Figure S2.5. Western blot analysis of selected MAPK kinases in BMDM infected with FSC200 wt or Δ*gapA* at MOI 50 for 15, 30 and 60 min. Data are representative from at least biological duplicate.

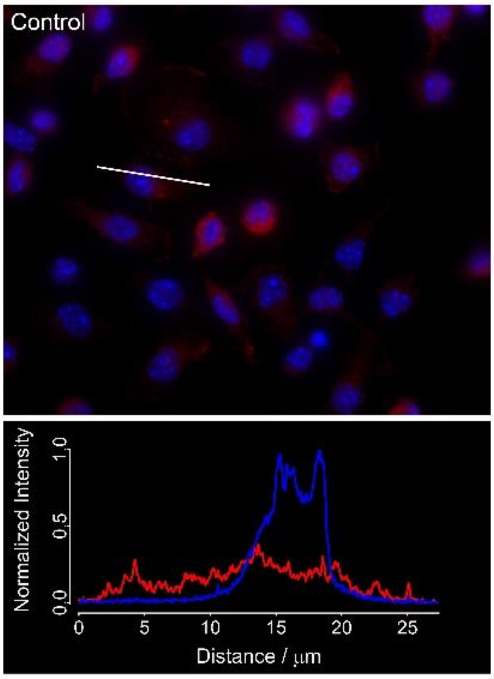

Figure S2.6. Fluorescence microscopy photomicrographs of fixed BMDM stained with Hoechst 33342 (blue; nuclei) and NF-κB p65 (red). Bar is indicating the measured part of an image for the fluorescence intensity profiles of both signals and represents 27 µm.

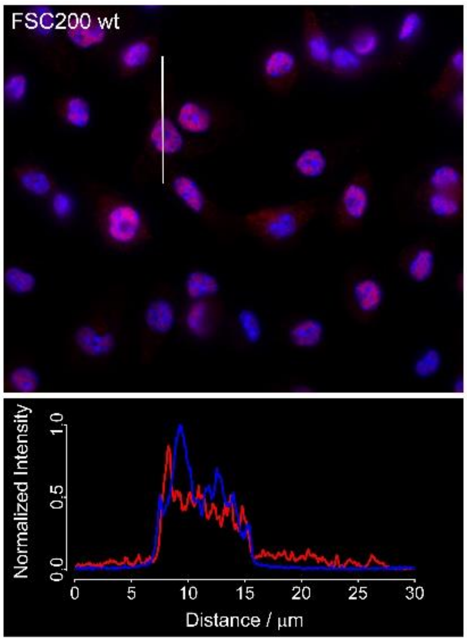

Figure S2.7. Fluorescence microscopy photomicrographs of fixed BMDM (infected with FSC200 wt) stained with Hoechst 33342 (blue; nuclei) and NF-κB p65 (red). Bar is indicating the measured part of an image for the fluorescence intensity profiles of both signals and represents 30 µm.

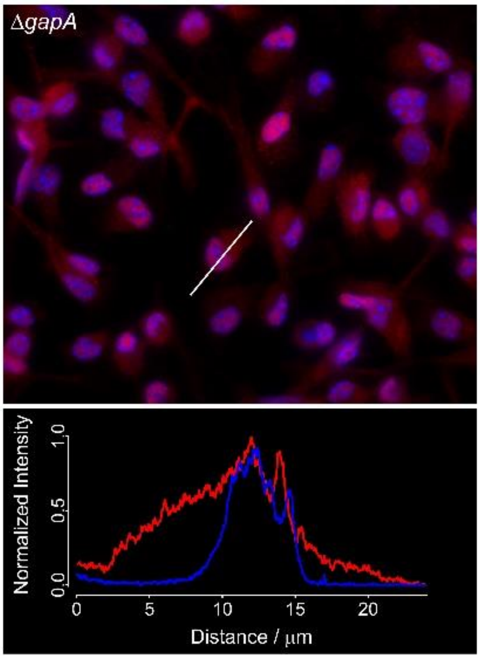

Figure S2.8. Fluorescence microscopy photomicrographs of fixed BMDM cells (infected with ΔgapA) stained with Hoechst 33342 (blue; nuclei) and NF-κB p65 (red). Bar is indicating the measured part of an image for the fluorescence intensity profiles of both signals and represents 24 μm.

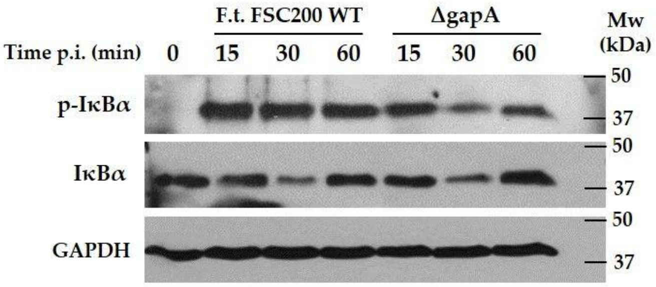

Figure S2.9. Western blot analysis of IκB-α in FSC200 wt and Δ*gapA* infected BMDM (MOI 50) during the first hour of infection. Data are representative from biological triplicate.

The IκB proteins form a complex with NF-κB transcription factors ensuring thereby their cytoplasmic localization in inactive state. Phosphorylation of IκBα results in its proteasome degradation and rapid translocation of released NF-κB transcription factors into the cell nucleus, where they promote expression of number of pro-inflammatory mediators as well as the expression of negative regulators incl. IκBα [1]. In BMDM infected with both the strains, phosphorylation of IκBα occurred 15 minutes i.p. and retained throughout the monitored period. Decreased amounts of IκBα due to degradation was obvious 30 minutes p.i. in cells infected with both strains.

[1] Rahman, M.M.; McFadden, G. Modulation of NF-KB Signalling by Microbial Pathogens. *Nat. Rev. Microbiol.* **2011**, *9*, 291–306, doi:10.1038/nrmicro2539.
